# Evidence-based Patient Choice and Consumer health informatics in the Internet age

**DOI:** 10.2196/jmir.3.2.e19

**Published:** 2001-06-07

**Authors:** Gunther Eysenbach, Alejandro R Jadad

**Keywords:** Consumer Health Informatics, Evidence-based medicine, Patient Education, Patient Participation, Physician-Patient Relations, Internet, Decision-Making

## Abstract

In this paper we explore current access to and barriers to health information for consumers. We discuss how computers and other developments in information technology are ushering in the era of consumer health informatics *,* and the potential that lies ahead. It is clear that we witness a period in which the public will have unprecedented ability to access information and to participate actively in evidence-based health care. We propose that consumer health informatics be regarded as a whole new academic discipline, one that should be devoted to the exploration of the new possibilities that informatics is creating for consumers in relation to health and health care issues.

## Introduction

For the past 100,000 years, people have been able to produce, distribute, and process information in a synchronized manner. About 500 years ago, the situation started to change rapidly. With the advent of the mobile typepress, our ability to produce and distribute information started to accelerate, outpacing our capacity to process information. During the past 10 years, we have witnessed how the Internet and the World Wide Web have led to a hyper-production and hyper-distribution of information, which have clearly overwhelmed our capacity to process it.

In this article we will explore current access to and barriers to further information for consumers. We will discuss how computers and other developments in information technology are ushering in the *era of consumer health informatics,* and the potential that lies ahead. It is clear that this will be a period in which the public will have unprecedented ability to access information and to participate actively in evidence-based health care.

We propose that consumer health informatics be regarded as a whole new academic discipline, one that should be devoted to the exploration of the new possibilities that informatics is creating for consumers in relation to health and health care issues. In its broadest sense, consumer health informatics should involve the following [1]:

- analysing, formalizing, and modelling consumer preferences and information needs;
- developing methods to integrate these into information management in health promotion, clinical, educational, and research activities;
- investigating the effectiveness and efficiency of computerized information, (tele)communication, and network systems for consumers in relation to their participation in health- and health care-related activities;
- studying the effects of these systems on public health, the patient-professional relationship, and society.

We will discuss the responses that are required of the health care professions and individual practitioners. There are also potentially helpful checks and balances on the nature of information now available to consumers. We will outline some of these and explore how all these developments may come together. None of these developments in information occur in isolation. They must be seen within the context of other changes, particularly the shifting emphasis away from the traditional paternalistic model of health care. These other changes are addressed more fully in other chapters of this book so will not be discussed in detail here. We will describe the development in information availability, but want the reader to place these issues in the broader context of moves towards greater informed choice for consumers in their health care decisions.

## Current access to information by consumers: the gap between the ideal and the real

Ideally (as long as they wish), all consumers should be able to access valid and relevant information about their health status. They should be able to judge the advantages and disadvantages of all possible courses of action, according to their values, beliefs, preferences, and their personal circum-stances (for example, their perceived state of health, their socio-economic status).

In reality, we are far from this ideal state, as many barriers prevent consumers from accessing the information they need, when they need it, where they need it, and in the amount and format in which they need it. The following is a brief description of some of the most prominent barriers. We do not pretend to include an exhaustive list, but a selection of those that, in our opinion, are preventing consumers from participating meaning-fully in evidence-based decision making. We have separated the barriers depending on whether they relate to providers, to the consumers *per se*,to the information available, to the health care system, and to information technology. As a theme in the titles of the following sections we will draw an analogy from the supply of water.

### Barriers related to providers: keeping the consumer thirsty

Despite a strong international trend to shift towards a shared decision making model, many consumers in both developed and developing countries still find themselves interacting with providers who favour the 'classical', authoritarian, paternalistic, asymmetrical model of consumer-provider interaction. In these situations, consumer access to information is prevented by health care providers who adopt the role of main purveyors of knowledge. The professional acts not only as the sole holder of the consumer's data but also as the filter for other types of information needed by the consumer to participate in decisions about their health and health care. In other cases, consumers face providers who prefer an 'informed choice' decision making model, in which they give consumers as much information as they think they need to make a decision, but the professionals do not participate directly in the decision. A shared decision making approach goes beyond this, placing consumers and providers as active participants in the decision making process, with two-way exchange of information and working as partners.

Even if providers wish to shift from the authoritarian or informed models to a shared one, however, many remain unable to do it because of inadequate communication skills, lack of time, or lack of financial incentives. A combination of the above factors may explain why many providers do not even think that consumers could benefit from the Internet. A survey from the US shows striking figures: only 39% of all professionals see the Internet as a valuable health information source for consumers. This sharply contrasts with the value consumers give to web-education: 70% of consumers retrieving health information on the Internet agree that 'the Internet empowers me to make better choices in my life' *(sourc* e: cyberdialogue/ findsvp survey, reproduced in Reents and Miller [2]).

Various factors probably contribute to the low esteem in which professionals hold the Internet as an educational tool. These include the (partly justified) concerns about the quality of Internet information and discomfort about having to deal with a consumer who is perhaps better informed than oneself. The Wilson study [3] illustrates the extent of this: an amazing 65% of the family doctors said that the information presented by consumers was new to them (see Table 1).

### Barriers related to consumers: a rocky road, few shoes and no maps to find the wells

#### Lack of easy-to-access sources of high-quality relevant information

Until very recently, databases such as Medline were available only to experts (sometimes not even to them). Although consumers were always 'passively' exposed to health information in the mass media, the possibilities to actively perform targeted literature searches were limited. Not only did consumers have limited insights into and access to the whole body of medical knowledge, but usually they also had virtually no access to their own medical records.

**Figure 1:**
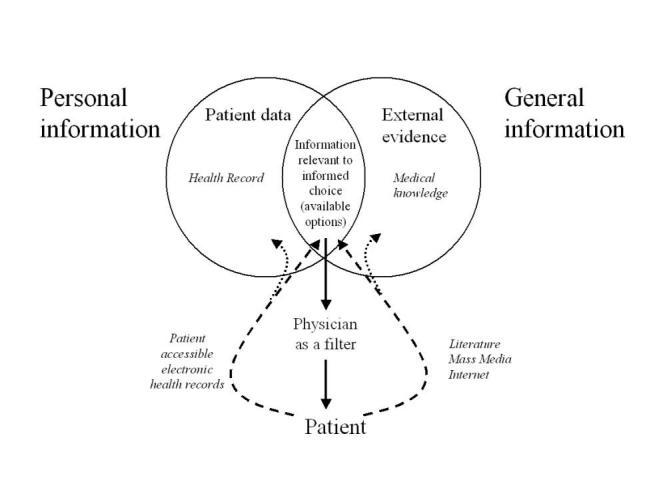
Consumer data and external evidence are the two categories of information that need to be integrated by the professional and consumer to arrive at a health care decision. Increasingly, consumers can bypass the professional as a filter (and moderator) and have direct access to parts of this information. This may be problematic, if the consumer accesses not only information that is relevant for their informed decision process, but also low-quality and irrelevant information. At the same time this is also an opportunity for evidence-based health care, as consumers are now able to question the evidence-base of professionals

**Figure 2:**
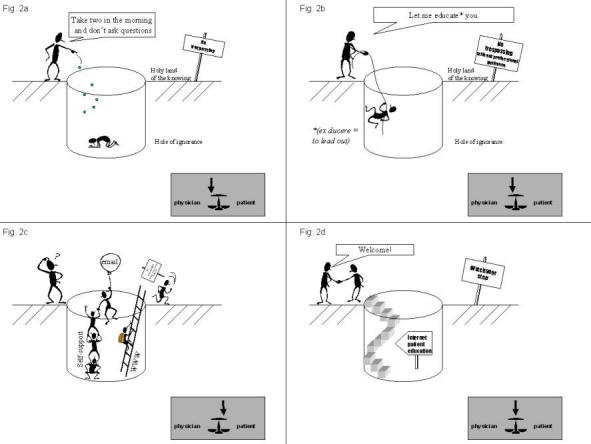
Different models of the consumer-professional relationship: a) paternalistic, b) educational, c) Internet-age, and d) consumer-as-partner

To date, it has been the 'traditional' responsibility of the professional to integrate all types of information in the personal interaction with the consumer. Thus they would give consumers details about their conditions and distil and present the relevant external information on the available options. Increasingly, however, the traditional professional - filter and sole provider of information - is being bypassed by consumers, who now have direct access to both the external evidence and their personal health record (Figure 1). This process is likely to accelerate and evolve quickly, thanks to powerful forces which are shaping health and health care, of which the Internet is perhaps the most prominent [4]. These changes are already facing resistance from the provider community. Many professionals are concerned that consumers may misinterpret information and will not arrive at the information that is relevant to them (intersection of Figure 1) but get lost in a stew of irrelevant and low-quality information. Vignettes of how the influence of information affects the models of care are illustrated in Figure 2.

**Table 1:** Survey among family doctors and practice nurses about consultations with consumers holding Internet health care information

	**Family doctor No. of Staff (%)**		**Practice Nurse No. of Staff (%)**	
The consumer participates more actively in his/her treatment	65	(78.3%)	26	(83.9%)
The consumer has higher expectations	75	(85.2%)	26	(78.8%)
The information is accurate	59	(73.8%)	24	(75%)
The length of consultation is increased	68	(77.3%)	24	(72.7%)
This type of consumer is a welcome challenge	46	(55.4%)	24	(72.7%)
The consultation is more interactive than usual	43	(50.6%)	22	(68.8%)
The consumer correctly interpreted information	38	(44.7%)	19	(59.4%)
The consumer is more demanding	50	(58.8%)	14	(42.4%)

#### The problem of low health literacy

Low health literacy frequently impairs consumers' understanding of health messages and limits their ability to care for their health problems [5]. This is a problem especially prevalent among the elderly [6]. Consumers with inadequate health literacy have a complex array of communications difficulties, which may lead to poor health outcomes. Individuals judged to be 'functionally illiterate' (estimated to include 30 to 50% of the adult population in the US and Canada) have been shown to report worse health status and have increased risk of hospitalization [7]. To compound this, much consumer education material has been produced which is at a higher reading level than the estimated average reading level of the American public [8] and most patient information on the WWW is written at even higher reading levels [9]. Unsurprisingly such material may fail to communicate the basic information intended.

Twentyfive years ago Tudor Hart [10] described the inverse care law, stating that 'the availability of good medical care tends to vary inversely with the need for it in the population served'. In analogy, we may postulate an *'inverse information la* w' [1] stating that access to appropriate information varies inversely with the need for it. In other words, it is likely that access to high-quality relevant information is particularly difficult for those who would need it most. At present, people with low health literacy do not benefit from advances in consumer health informatics and cybermedicine, as they lack access to or understanding of these technologies. A sequence can be envisaged in which low health literacy leads to poor health, poor health leads to low income, and low income limits access to modern information technology. Thus, one fundamental problem of telemedicine and using the Internet for health education is that those who are at highest risk of preventable or treatable health problems have the greatest need for information and are the least likely to have access to such technologies [11].

Public policies are needed to actively fight this pervasive inequality. It is also important to realize that there needs to be greater awareness about the problem of health literacy. The American Medical Association's Ad Hoc Committee on Health Literacy for the Council on Scientific Affairs concluded that 'professional and public awareness of the health literacy issue must be increased, beginning with education of medical students and professionals and improved consumer-professional communication skills' [5]. In addition to efforts to increase awareness, we also need to develop better methods of screening consumers to identify those with poor health literacy, more effective health education techniques, and more research on outcomes and costs associated with poor health literacy, and the causal pathway of how poor health literacy influences health.

#### Limited access to the Internet

Even if there were resources that provided high-quality information easily on the Internet, regardless of their literacy levels, a major barrier that would still need to be overcome is the broader barrier of access, described in detail below ('Barriers related to technology'). Thus, the inverse information law is true both on the macrolevel - the poorest countries have the worst access to information and communication [12] - and on the individual level (microlevel), with disadvantaged individuals within a society having the poorest health, inferior health literacy, and the worst access to information.

### Barriers related to the information: hydrants with muddy water

#### Unlimited access to poorly organized information

In the past, health professionals had to cope with information overload, while consumers had to cope with information deficit. Today, consumers have many opportunities to access information in abundance, through mass media, self-support groups, and particularly the Internet (Figure 2c). The directed, intentional process of active 'health education' (Figure 2b) is now being counteracted by an anarchical process of uncontrolled information retrieval by the consumer.

For the first time in the history of medicine, consumers have equal access to the knowledge bases of medicine - and those 'connected' are making heavy use of this. An example of this is the fact that the number of Medline searches performed by directly accessing the database at the National Library of Medicine increased from 7 million in 1996 to 120 million in 1997, when free public access was opened. The new searches are attributed primarily to 'non-professionals' [13]. It has been argued that 'a driving force behind demand for online health information is the shortage of information easily obtained from traditional channels' [2]. With the duration of an average consultation still only seven minutes in the UK (and twelve minutes in the US) it comes as little surprise that professionals routinely fail to address the information needs of consumers [4]. While most professionals do not understand or have access to these modern information technologies, or simply lack sufficient time to familiarize themselves with the Internet, consumers have all the time in the world to search the Internet for relevant information.

This new 'reversed' information asymmetry creates new conflicts - the fact that consumers are taking the initiative to look out for the latest research results 'stands on its head the tradition in which a doctor gives orders and the consumer obeys', as an article in the *New York Times* put it. 'And that makes some doctors nervous' [14]. Some of the concern is well founded. It is likely, for instance, that health professionals may find themselves in the middle of unnecessary conflicts if consumers find information on the Internet that is unknown to the professional, contradicts their recommendations, or that suggests the use of an effective intervention that is unavailable.

In a postal questionnaire survey among 160 family doctors and 96 practice nurses in Scotland [3], 58% of doctors and 34% of nurses stated that they have been approached by consumers with Internet health care information. Only 39% of the doctors and 31% of the nurses felt 'positive' about these consumers, the remainder were 'indifferent', 'uncomfortable', or 'not sure'. About half of the respondents were concerned about the reliability of Internet information and a similar percentage were concerned that consumers did not interpret information correctly [3]. On the positive side, the majority of health professionals feel that when consumers bring information they participate more actively in their treatment, that the consultation is more interactive, and that overall 'this type of consumer is a welcome challenge' (see Table 1).

The almost unlimited access to information offered by the Internet also creates other potential problems. Seeking desired information on the Internet is often time-consuming. Consumers often experience confusion and anxiety caused by the virtually unlimited amount of information available, which is poorly organized and has quite variable quality and relevance.

#### Few mechanisms to control the quality of the information

Currently there is no agreed mechanism for ensuring the accuracy, currency, or completeness of the information presented to consumers [15]. A quality control process, both when preparing information and when accessing it, has been demanded from different sides. A recent review of 54 consumer information materials concluded that 'current information materials for consumers omit relevant data, fail to give a balanced view of the effectiveness of different treatments, and ignore uncertainties; moreover, many information materials adopt a patronizing tone - few actively promote a participative approach to decision making' [16].

On the Internet, there have been numerous studies evaluating the quality of information given on different venues such as websites [17], newsgroups [18] and email-consultations [19,20]. While the Internet offers a huge amount of health information, many of the authors are not trained in medicine or even health education. In many situations, the intention of information provision is not to educate, but to sell something.

The lack of reliability is a particular concern. In addition to this, the Internet poses special problems for consumers, which have been summarized as 'lack of context' [21], meaning that the Internet poses additional problems for consumers and health professionals to assess and apply the material, compared to critical appraisal of traditional information. This is due to the following characteristics of the Internet [22]:

- There are no clear markers such as traditional publishing which allow consumers to recognize:
  1. the target group of a document (consumers/professionals)
  2. the intention (advertisement or objective information);
- The anonymity (of authors) makes it difficult to appraise information based on the credentials of the authors;
- Internationality: information valid in foreign health care systems may not be applicable locally [23].

These characteristics of the Internet may explain why consumers have difficulties finding information that relates to them and why the majority of physicians say that the consumer has difficulties interpreting information correctly [3]. While it has been pointed out that we still know very little about the impact of the Internet on public health [24], there are many ways that Internet information could do harm [25]:

- Misinformation can lead consumers with life-threatening conditions to lose trust in their provider, and take actions that undermine the effectiveness of their treatment (such as by taking substances that interact in a negative way with prescribed medications).
- Consumers may use their limited time with their health care provider unproductively, or in ways that ultimately increase costs of care, and even abandon a provider delivering high-quality care to pursue ineffective therapies.
- Vulnerable people may also be victimized by biased or incomplete information from those with a financial interest in the information they provide.

Such risks are present in most media, but on the WWW this problem reaches a new dimension.

### Barriers related to technology: few pipes, few glasses, and complex taps

If consumers are to take full advantage of the Internet, access to it should be easy, affordable, and available in all settings. This is still far from reality. Despite an unprecedented rate of penetration in developed countries, the majority of people in the world remain without access to computers and the Internet. The Internet is still available to less than 50% of people in North America, the region with the highest proportion of users in the world. In developing countries, the main barriers are the high cost of computers and poor telecommunications infrastructure. In both developed and developing countries, many consumers still perceive computer-based systems as difficult to use.

The end result is that rather than levelling the playing field, the rapid development of the Internet is contributing to widening inequalities across the world [26]. Even in developed countries, there is some evidence of a similar widening gap across groups with different socio-economic and demographic profiles [12]. There is a clear digital divide between the information rich (such as Whites, Asians/Pacific Islanders, those with higher incomes, those more educated, and dual-parent households) and the information poor (such as those who are younger, those with lower incomes and education levels, certain minorities, and those in rural areas or central cities) [27]. The levels of access appear to be increasing rapidly in other parts of the world, particularly in Western Europe and in the developed countries of Australasia. Although the data are very poor, it seems that the developing world is lagging behind, creating an increasingly wide access gap.

While the information society offers tremendous potential for reducing the knowledge gap between professionals and patients, it also brings a risk of a widening of the gap between those who have access to new technology and those who have been excluded. Therefore the field must not be left to market forces alone and active policy is required to push information technology to those who are underserved [1].

## Striving for the ideal: bridging the gaps through information technology

### Developing advanced approaches to knowledge representation

So far, most (if not all) of the Internet-based applications to promote transfer of knowledge to consumers are a mere transition from paper-based to electronic-based means to process and distribute information in text form. The true 'revolution' (in the sense of going full circle), however, is likely to come from ongoing and future increases in bandwidth that will enable all people to communicate through the Internet more effectively. The next generation Internet (see www.ngi.gov) will operate at speeds up to a thousand times faster than today. Sight, sound, and even touch will be integrated through powerful computers, displays, and networks. With these developments we will be able to go beyond text to more 'natural' or primal ways of representing and exchanging knowledge. Soon we will be able to put together and deliver relevant and valid information, of different types, using more engaging ways to package the messages and multisensory modes of communication. The effectiveness and efficiency of these new modalities to organize information will be optimized through inexpensive Internet appliances (such as fridges and microwave ovens with Internet access), personal portable or wearable computers, and wireless access to the Internet [28].

Another trend will lead to a 'quality leap': the perspective of 'machine understandable information'. Key to this development is the widespread use of metadata. Recent developments and Internet standards, such as the eXtensible Markup Language (XML), Dublin Core metadata [29], MedPICS [21,22], and RDF (Resource Description Framework), will make relations between information pieces 'understandable' for computers, allowing software for example to perform intelligent searches, filter information automatically, or to tailor information to the individual. The Web would evolve into a global medical knowledge base that is easily navigable and searchable across languages and continents [30].

### Promoting team work

It is time for health professionals 'to embrace the concept of informed consumers and use their web-surfing skills' [31] (see also Table 2) rather than seeing them as threatening intruders trespassing into a forbidden zone. For the providers, this requires the acquisition of skills in the use of the Internet, familiarization with sources of high-quality information [32], and confidence with the use of aids and tools to engage in shared decision making. On a public health level, 'stairways' for the consumer should be built, guiding consumers to high-quality information, as illustrated in Figure 2d. Examples include 'Healthfinder', a government-sponsored health portal in the US (www.healthfinder.gov) or the National electronic Library for Health (NeLH) in the UK. The latter's mission is 'to improve health and health care, consumer choice, and clinical practice', and it includes NHS Direct Online, a service to provide consumers with information such as 'How can I stay healthy, feel better, and reduce the risk of disease?', 'Do I need to see a doctor for this problem?', and 'How can I learn more about my condition, contribute to my care, and make the best use of health services?' [33]. Clearly, the demand for such information is vast, as can be seen in the large number of patient requests doctors on the Internet receive via email [34,19,35].

**Table 2:** Suggestions for providers to interact with Internet-literate consumers

**Do** Try to react in positive manner to information from the Internet Warn about the variability in the quality and reliability of material from the Internet Warn about time constraints that may limit your ability to address all the information found on the Internet Develop a strategy for dealing with Internet information before the encounter (e.g. get consumers to email summary of issues before consultations) Accept consumer contributions as valuable Accept that they may find relevant and valid information previously unknown to you.
**Don't** Be dismissive or paternalistic Be derogatory of comments made by others on the Internet Refuse to accept information found on the Internet Feel threatened

## Giving consumers control over their own information

One of the most radical steps towards consumer empowerment will involve making the electronic health records (at least parts of them) available to consumers on the Internet. Once this occurs, consumers will be able to do 'online-doctoring', just as they do 'online-banking' and 'online-shopping' today [1].

To put the records into the hands of consumers is not a new idea. More than 25 years ago it was already advocated that 'patients' should be able to take their records home [36]. Baldry [37] conducted an early experiment with giving consumers in the waiting room their medical records to read. The international trend is to allow consumers to inspect their records and to allow them to make copies there of [38]. The European Union Data Directive (applicable October 1998) required all member countries to enact legislation enabling subject access to medical records, if not already enacted.

Consumer health informatics developments offer further opportunities for this process, with the potential to grant consumers access to information which is relevant to them and to integrate their personal data with explanatory information. For example, a system called SeniorMed allows elderly consumers access to their electronic medication lists via the WWW. Such systems may be integrated with drug information [39]. MedicaLogic, a company based in the US, is also testing a concept called 'Internet Health Record', a service that lets consumers privately access information from their real medical records over the Internet. The information is embedded in a system that lets them research health conditions, refill prescriptions, and communicate with their professional's office. Consumer records could be linked with glossaries, and be linked to information on the Internet (for example, if the problem list contains 'smoking', links could refer to 'how-to-quit-smoking' health promotion sites or to Medline). Consumers could also change or comment on certain entries (http://www.medicalogic.com/services/about_98point6.html).

**Figure 3:**
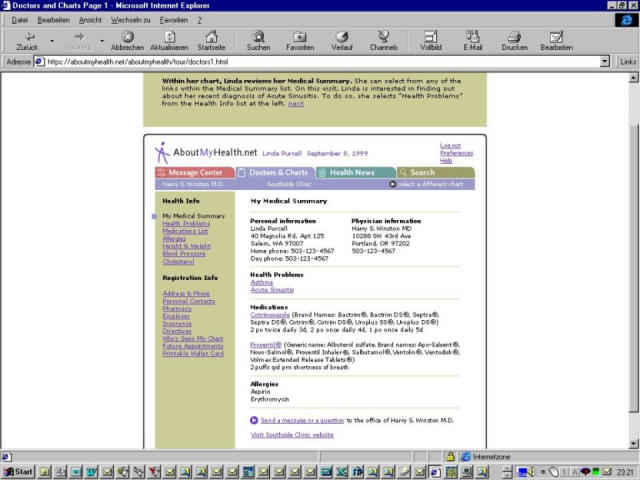
Internet accessible consumer health record

## Ensuring better quality control

In the field of Internet publishing, many instruments to evaluate health information exist, but none of them have been validated. In addition, it is unclear whether they should exist in the first place, whether they measure what they claim to measure, or whether they lead to more good than harm [40].

The Swiss Health on the Net foundation has compiled some consensus ethical principles for publishers of health information, the so-called HON Code of Conduct (http://www.hon.ch/HONcode/). Information providers who agree to implement these ethical principles display the HON logo on their websites. However, there are no mechanisms for controlling or enforcing the adoption of such principles. As a result, it is not clear how many of the several thousand sites displaying the logo have actually implemented the principles. The HON Code is often misinterpreted (also in the peer-reviewed literature!) as an award-system, rating system, or as 'quality criterion' which allows consumers to appraise the quality of a website. It is however not possible for a third party (that is, the user of a website) to verify for example that a principle such as 'privacy and confidentiality' or 'honesty in disclosing sources of funding' is observed.

A systematic review on different quality criteria used to assess information on the Internet has been published recently [41]. Consumers may for example use indirect quality criteria such as popularity, expressed as number of visitors or 'webcitations' [21,42]. There are now several tools available on the Internet for use by consumers which help users to assess the quality themselves (http://hitiweb.mitretek.org/iq/default.asp, http://www.discern.org.uk, http://www.quick.org.uk).

DISCERN is a standardized index to judge the quality of health information. This instrument is targeted at producers, health professionals, and consumers to appraise written information on treatment choices. Crucial in the development was the determination of inter-rater agreement among different user groups. Questions with insufficient inter-rater agreement, such as those concerning design or reading level ('the information is easy to understand'), were eliminated from the final instrument. However, the validity of DISCERN in terms of the relationship between a DISCERN score and impact of the information on consumer outcomes have not yet been determined. It should also be noted that the inter-rater reliability for DISCERN was rather low when it was used by consumers. Thus it is not yet clear whether DISCERN is a truly useful instrument for consumers to distinguish good from bad information. In the near future, an international system of accreditation or 'quality seals' (evaluative meta-information assigned by trusted raters) may help consumers to identify high-quality information on the Internet. A European Union (EU) funded project, 'G7 ENABLE', has described 'Barriers To A Global Information Society For Health'. It made the following observations to the EU Commission: 'A great deal of health-related information on the Web is poor, misleading and much positively harmful. This substantially diminishes the benefits that the Internet could potentially deliver'.

What is required is an internationally-recognized scheme whereby the public can identify, and search for, high-quality Internet health information. These should carry the authority of clinical bodies, which are recognized as having the clinical standing to be trusted. Such a filtering and rating system is currently being implemented in a new EU project called MedCERTAIN (MedPICS Certification and Rating of Trustworthy Health Information on the Net, http://www.medcertain.org), funded under the EU Action Plan for safer use of the Internet [43]. The aim of this project is to establish trust and improve the quality of health information on the Internet by the 'four E's' [44]:

- *Educating* the public (teaching critical appraisal skills to consumers);
- *Encouraging* self-governance, for example encouraging health information providers to obey ethical codes for health [44] and promoting self-labelling (disclosure of important information such as authorship and sponsors, also with metadata);
- *Evaluation* and certification of information (offer a framework for third party rating, so that interested medical societies and bodies can assign 'quality seals' to trustworthy information)
- *Enforcement*(Network of Hotlines for consumers)

The international MedCERTAIN trustmark will be established in close collaboration with all interested agencies and relevant national organizations which pursue similar aims. These would include, for example, bodies such as OMNI (Organizing Medical Networked Information, http://www.omni.ac.uk/) or the UK Centre for Health Information Quality. A basic principle is inter-operability of existing rating services and the creation of metadata exchange standards.

## The future

The vast potential of the Internet to promote health information and to foster consumer-professional communication is far from being realized. The Internet has both the clientele (consumers who really want to learn something about their health) and the technical prerequisites (the reach of a mass-medium, combined with the possibility for interactivity to tailor information specific to the individual) to be an ideal medium to promote consumer education and decision support. An interesting future perspective is the linkage of the personal online-accessible health record with general health information from evidence-based resources. The convergence of technology and knowledge will be greatly enhanced by the use of multimedia and artificial intelligence. Further contributions will come from the advent of low cost portable and wearable computers. These will allow access to knowledge at the right time and in the right place through ubiquitous computer networks and wireless connections to the Internet. Among other challenges [45], development and proper evaluation of these tools and making them accessible to those who need them most will be the main themes of consumer health informatics in the information age.
